# Effect of fat deposition on placental function in Shaziling sows and modulation by resveratrol

**DOI:** 10.1016/j.aninu.2025.08.009

**Published:** 2025-12-06

**Authors:** Xizi Yang, Ruizhi Hu, Wentao Zhang, Mingkun Shi, Zhiyong Fan, Xi He, Chenxing Fu, Liang Chen, Hongfu Zhang, Xupeng Yuan, Maisheng Wu, Yulian Li, Hong Tan, Jianhua He, Shusong Wu

**Affiliations:** a Hunan Collaborative Innovation Center for Utilization of Botanical Functional Ingredients, College of Animal Science and Technology, Hunan Agricultural University, Changsha 410128, China; b State Key Laboratory of Animal Nutrition, Institute of Animal Sciences, Chinese Academy of Agricultural Sciences, Beijing 100081, China; c College of Animal Science and Technology, Hunan Biological and Electromechanical Polytechnic, Changsha 410127, China; d Animal Breeding Station of Xiangtan, Xiangtan 411104, China

**Keywords:** Fat deposition, Backfat thickness, Resveratrol, Shaziling sow, Placental function, Angiogenesis

## Abstract

Excessive fat deposition is considered as an important factor impairing reproductive performance in sows, and resveratrol (RES) has shown a hypolipidemic effect in previous studies. This study aimed to investigate the impact of fat deposition on reproductive performance of Shaziling sows, and further clarify the intervention by RES. Fifty-six lard-type Shaziling sows at 75 d of gestation (G75d) were divided into four groups (*n* = 14) based on backfat thickness (BT): backfat thickness between 20 to 24 mm (NBT) and backfat thickness between 26 to 30 mm (HBT). The NBT-CTL and HBT-CTL groups were fed a basal diet, while NBT-RES and HBT-RES were fed a diet containing 500 mg/kg RES until delivery. Results showed that HBT sows had lower total litter weight (*P* = 0.008), average weight of total births (*P* < 0.001), live litter weight (*P* < 0.001), average weight of live birth (*P* < 0.001), and placental efficiency (*P* = 0.013), but higher average process of farrowing (*P* < 0.001) and stillbirth (*P* = 0.005), while RES recovered these indicators significantly. In addition, HBT sows showed lower placental vascular density with decreased fluorescence intensity of platelet endothelial cell adhesion molecule-1 (*CD31*; *P* = 0.006) and fibroblast growth factor (*FGF*; *P* = 0.001), and expression of nutrient transport genes including sodium coupled neutral amino acid transporter 1 (*SNAT1*; *P* = 0.029), cluster of differentiation 36 (*CD36*; *P* = 0.004). RES increased nutrient transport genes including glucose transporter protein 3 (*GLUT3*; *P* < 0.001), *GLUT4* (*P* = 0.014), cationic amino acid transporter 1 (*CAT-1*; *P* = 0.005), *SNAT2* (*P* = 0.015), and plasma membrane fatty acid binding protein (*FABPpm*; *P* = 0.009), but increased placental vascular density by increasing the expression of placental angiogenesis (*FGF*; *P* = 0.013), placental growth factor (*PlGF*; *P* < 0.001) and vascular endothelial growth factor (*VEGF*-*A*; *P* = 0.013) in placenta. Additionally, RES recovered the phosphorylation of phosphoinositide 3-kinase (PI3K; *P* < 0.001), protein kinase B (AKT; *P* < 0.001), and mammalian target of rapamycin (mTOR; *P* < 0.001) in placenta of HBT sows. These findings suggest that excessive fat deposition can impair reproductive performance, which is rescued by RES by increasing the expression of placental nutrient transport and angiogenesis genes in lard-type Shaziling sows, and PI3K may play an important role.

## 1 Introduction

Shaziling pigs are a local pig breed in Hunan in China, belonging to one of “Central China Two-end-black Pigs”, with superior meat quality, coarse feed tolerance, and strong adaptability, which is one of the local signs of Chinese agricultural products (Chen et al., 2022, 2021). However, Shaziling pigs are a lard-type breed, which have a slow growth rate, high fat rate, thick backfat, and a low birth weight of piglets (Song et al., 2022). According to incomplete statistics, the litter size of Shaziling sows is 12.39, and the litter weight (7.59 kg), and individual average weight (0.87 kg) are relatively low, which cause its restricted utilization on commercial farms referring to Hunan Province Livestock and Poultry Variety Catalogue and Variety Map (1984). Previous study in lean type sows demonstrated that there is a significant quadratic effect between litter weight and backfat thickness (BT) (Zhou et al., 2018). High BT of sows causes lower numbers of piglets born alive per litter and litter birth weight (Hu et al., 2022). Excessive fat deposition of maternal exacerbates metabolic syndrome and insulin resistance in late pregnancy (Wu et al., 2023a), increases triglyceride content in placenta, causing ectopic deposition of placental fat, leading to placental dysplasia and dysfunction (Saben et al., 2014), thereby reducing sow reproductive performance, including less litter size (Kim et al., 2015), a greater proportion of intrauterine growth restriction (IUGR) (Zhou et al., 2018), and lower litter weight (Cheng et al., 2019). However, the above studies focused on lean type sows, there is rare research on the relationship between BT of fat type sows, such as Shaziling sows, and reproductive performance.

The placenta is the singular fetal tissue in direct contact with the maternal environment, and the main function is as a unique medium for nutrient absorption for the fetus from the maternal bloodstream and allows release of metabolic wastes to the mother (Huang et al., 2021; Wu et al., 2022). Placental angiogenesis is regulated by relevant regulatory factors, for facilitating optimal transport of nutrients, oxygen, and metabolic byproducts between the mother and fetus. Studies demonstrated that the length, surface, volume, and density of the placental villi and capillaries in IUGR fetuses decrease (Chen et al., 2002; Mayhew et al., 2004), indicating that placental angiogenesis is an essential factor in determining fetal survival and growth. High backfat sows had lower placental vascular density, vascular endothelial growth factor (VEGF-A), cell adhesion molecule-1 (CD31) protein expression (Wu et al., 2023b), and phosphorylation of vascular endothelial growth factor receptor 2 (p-VEGFR2) (Hu et al., 2019). Excessive fat deposition also inhibits the invasion of trophoblast, affecting placental development, lipid metabolism, and transport, thereby influencing fetal developmental pathways (Yang et al., 2023).

Resveratrol (RES) as a natural non-flavonoid polyphenol stilbene, possesses multiple biological activities, exists in various plants including grapes, berries, and peanuts. It also could be extracted from *Polygonum cuspidatum* (Meng et al., 2023). Our previous study in lean meat pigs indicated that 300 mg/kg RES increased the placental vascular density and nutrient transporter genes expression of sows (Hu et al., 2022). Supplementary RES to the diet of sows increased litter weight (Meng et al., 2018). Moreover, RES has been shown to have regulatory effects of fat and energy metabolism (Gimeno-Mallench et al., 2019; Hsu et al., 2020). Therefore, this study aims to use two-factor experimental design to explore the effect of BT on reproductive performance and placental function of Shaziling sows, as a lard-type breed, and further explore the regulatory mechanism after supplementation with RES.

## 2 Materials and methods

### 2.1 Animal ethical statement

The animal experiment accepted supervision and inspection from the Biomedical Research Ethics Committee of Hunan Agricultural University (approval number 20240331).

### 2.2 Animals, diets, and experimental design

This experiment was conducted at the Shaziling Pig Breeding Farm in Xiangtan (Hunan, China). According to the BT of Shaziling sows at 75 d of gestation (G75d), 56 were selected with a parity from five to eight and the pigs were then divided into four groups (*n* = 14): BT between 20 to 24 mm (NBT-CTL), BT between 20 to 24 mm (NBT-RES), BT between 26 to 30 mm (HBT-CTL), and BT between 26 to 30 mm (HBT-RES). The NBT-CTL and HBT-CTL groups were fed a basal diet, while NBT-RES and HBT-RES were fed a diet containing 500 mg/kg RES (the dosage of RES is based on the research results of others and the previous study of our group). Sows were housed separately in gestation stalls with free access to water. Feed was provided daily at 07:00 and 17:00, at a total of 2.4 kg/sow per day. The basal diet composition based on the GB/T 39235-2020 (China National Standard, 2020) as shown in Table 1. RES (≥98%) was provided by Hunan Engineering and Technology Center for Natural Products (Changsha, Hunan, China). The experiment started on G75d and ended at the day of delivery.

**Table 1:** Composition and nutrient levels of diets (%, DM basis).

Item	Content
**Ingredients**	
Corn	41.90
Soybean meal	5.30
Wheat flour	20.00
Rice bran meal	5.80
Wheat bran	17.00
Beet meal	4.00
Soybean husk	2.50
Limestone	1.30
Rice husk powder	0.22
Propionic acid (50%)	0.12
Phytase	0.05
NaHCO_3_	0.30
Sodium chloride	0.40
L-Lys HCl (70%)	0.47
L-Thr	0.12
Choline chloride	0.15
DL-Met	0.07
Premix^1^	0.30
Total	100.00
**Nutrient levels**	
NE, MJ/kg	9.20
CP	13.50
Crude fiber	6.30
Organic matter	94.85
Calcium	0.65
Total phosphorus	0.55
SID Lys	0.65
SID Met	0.24
SID Thr	0.44
SID Trp	0.12
SID Val	0.45

DM = dry matter; NE = net energy; CP = crude protein; SID = standardized ileal digestibility.

1 The premix provides following for per kg diet: vitamin A, 9920 IU; vitamin B_1_, 2.2 mg; vitamin B_2_, 10 mg; vitamin B_3,_ 44 mg, vitamin B_5_, 33 mg; vitamin B_6_, 3.3 mg; vitamin B_7_, 220 mg; vitamin B_9_, 1325 mg; vitamin B_12_, 37 mg; vitamin D_3_, 1985 IU; vitamin E, 66IU; vitamin K, 4.4 mg; Cu, 8 mg; Fe, 80 mg; Mn, 45 mg; Zn, 80 mg; I, 0.5 mg; Se, 0.3 mg.

### 2.3 Sample collection

On the G75d and the day of delivery, the BT was measured at the outer tangent of the last rib 6.5 cm away from the midline of the back (P2) point via ultrasound which was performed by the same person throughout the trial. On the day of delivery, farrowing time, numbers of total births and stillbirths, piglet body weight, placental weight, and placenta efficiency (litter body weight/placental weight) were recorded, and blood and placental samples of all sows were collected, with placenta tissues selected 10 cm away from the umbilical cord. A portion of fresh placental tissue from each sow was fixed in 4% paraformaldehyde for further research, and the remaining stored at −80 °C. Blood samples stood at room temperature for 30 min, then centrifuged at 1500 × *g* (10 min), the serum obtained and stored at −80 °C.

### 2.4 Serum biochemical indexes

Assay kits (Nanjing Jiancheng Bioengineering Institute, Nanjing, Jiangsu, China) were used to measure levels of total cholesterol (TC; A111-1-1), triglyceride (TG; A110-1-1), low-density lipoprotein (LDL; A113-1-1), high-density lipoprotein (HDL; A112-1-1), and glucose (F006-1-1) levels in serum. The serum level of malondialdehyde (MDA; AKFA013M) and total antioxidant capacity (T-AOC; AKAO012M) were measured using assay kits purchased from Beijing Boxbio Science & Technology Co., Ltd. (Beijing, China); serum insulin (SEKP-0008) level analyzed using an assay kit from Beijing Solarbio Technology Co., Ltd. (Beijing, China); and an assay kit from Jiangsu Meimian Industrial Co., Ltd. (Yancheng, Jiangsu, China) to measure levels of tumor necrosis factor-α (TNF-α; MM-0383O1), interleukin(IL)-1β (MM-0422O1), and IL-6 (MM-0418O1) in serum. All indicators were measured using Varioskan Flash (Thermo Fisher Scientific Inc., Waltham, MA, USA) at the required wavelength after reagent reaction.

### 2.5 Histology

The placental tissues were submitted to Wuhan Servicebio Technology Co., Ltd. (Wuhan, Hubei, China) to stain with haematoxylin and eosin (H&E) and oil red O, respectively. Briefly, placenta tissue was fixed in formalin overnight at 4 °C. Then titrated with 50% ethanol for dehydration, paraffin embedded, sectioned, and slices put into xylene Ⅰ and xylene Ⅱ for 20 min individually, absolute ethanol Ⅰ and absolute ethanol Ⅱ for 5 min individually, soaked in 75% ethanol for 5 min, then washed with tap water. The slices were stained with H&E dye solution and sealed with rhamsan gum. Finally, microscopy and image acquisition was performed using MShot Biological Microscope ML31.

Oil red O: Mixed six parts of saturated oil red O dye solution with four parts of distilled water thoroughly overnight at 4 °C. Filtered once with qualitative filter paper, placed at 4 °C for 24 h, then filtered for the second time to obtain the oil red O working solution. Slices were immersed in oil red staining solution for 8 to 10 min (covered to avoid light). Then removed and let stand for 3 s before immersing in two cylinders of 60% isopropanol for differentiation, each for 3 and 5 s. Slices were sequentially immersed in two cylinders of pure water for 10 s. The slices were then removed and allowed to stand for 3 s, then immersed in hematoxylin for 3 to 5 min for re-staining. Slices were then immersed in three cylinders of pure water for 5, 10, and 30 s each, then differentiation solution for 2 to 8 s, washed with distilled water in 2 cylinders for 10 s each, and turned back into the blue solution for 1 s. The slices were then gently immersed in 2 cylinders of tap water for 5 and 10 s each, and the staining effect was observed under a microscope. Glycerol gelatin sealing agent was used for sealing. Microscopic examination, image acquisition and analysis was then performed.

### 2.6 Immunohistochemical staining

The placental tissues were fixed in formalin overnight for immunohistochemical staining. Briefly, paraffin embedded, sectioned, deparaffinated (65 °C for 2 h, xylene (Ⅰ) for 10 min, xylene (Ⅱ) for 10 min, anhydrous ethanol (Ⅰ) for 5 min, anhydrous ethanol (Ⅱ) for 5 min), hydration (95% ethanol for 5 min, 85% ethanol for 5 min, 75% ethanol for 5 min, wash with phosphate buffered saline (PBS) for 5 min 3 times, washed with Tris buffered saline (TBS) for 30 s, and antigen repaired (slices were placed in high-pressure ethylenediaminetetraacetic acid [EDTA] antigen repair buffer for 1600 W high-pressure repair for 3 min, placed in PBS and washed three times on a decolorization shaker for 5 min, and washed with PBS for 5 min repeated two times). The circle was sealed with serum, the sealing solution was shook off, and then incubated overnight with antibodies at 4 °C. The slides were washed three times (each time 5 min), then covered with the secondary antibodies corresponding to the first antibodies dropwise into the circle, and incubated in the dark for 50 min at room temperature. Next, nuclei were counterstained with 4',6-diamidino-2-phenylindole (DAPI), quenched with tissue autofluorescence, slightly dried and sealed with an anti-fluorescence quenching sealing agent. Finally, microscopic examination was performed. Antibodies were sourced from AiFang Biological (Changsha, Hunan, China). The dilution ratio of antibodies is as follows: CD31 (SAF005) 1:500, p-p50 (AF00666) 1:1000, p50 (AF02889) 1:200, p-p65 (AFWP0124) 1:200, p65 (AF300096) 1:100, VEGFR1 (AF02221) 1:300, VEGFR2 (AF02593) 1:300, FGFR1 (AF04718) 1:1000, p-VEGFR1 (AF00287) 1:300, p-VEGFR2 (AF00220) 1:300, p-FGFR1 (AF01134) 1:1000, phosphoinositide 3-kinase (PI3K; AFW6688) 1:1000, protein kinase B (AKT; AFW5523) 1:500, mammalian target of rapamycin (mTOR; AFW2445) 1:1000, p-PI3K (AF00823) 1:1000, p-AKT (AF00259) 1:500, p-mTOR (AF003535) 1:1000. CD31 and p65 are monoclonal antibodies. P-p65, p-p50, p50, VEGFR1, VEGFR2, FGFR1, p-VEGFR1, p-VEGFR2, p-FGFR1, PI3K, AKT, mTOR, p-PI3K, p-AKT, and p-mTOR are polyclonal antibodies.

### 2.7 Real-time quantitative PCR (RT-qPCR)

Real-time quantitative PCR assays were validated according to the MIQE guidelines (Bustin et al., 2009). The mRNA expression of *GLUT1*, *GLUT3*, *GLUT4*, *SNAT1*, *SNAT2*, *CAT-1*, *FABPpm*, *hFABP*, *CD36*, *TNF*-α, *IL-1β*, *GPX-1*, *GPX-4*, *SOD-1*, *SOD-2*, *VEGF-A*, *FGF*, and *PlGF* in placenta of Shaziling sows were determined by RT-qPCR. Tissue mRNA was extracted from placenta using a Steady Pure universal RNA extraction kit (AG21017), and RNA degradation and contamination was examined by electrophoresis. NanoDrop lite (Thermo Fisher Scientific Inc., Waltham, MA, USA) used for quantifying concentration of RNA. Reverse transcription (1 μg total RNA) using the Evo M-MLV kit (AG11707), fluorescence quantification using Taq-HS SYBR Green Premix Pro qPCR kit (AG11740). The three kits were purchased from Accurate Biotechnology Co., Ltd. (Changsha, Hunan, China). The above operations are strictly in accordance with the instructions of the kit determination. The parameters of thermal cycler as follows: 95 °C 3 min, then 95 °C 5 s, and 60 °C 30 s for 40 cycles. By measuring the fluctuation range of the Ct values, evaluated the stability of the β-actin and *GADPH* genes. Using 2^−ΔΔCT^ method to analyze data. A standard melting curve was used to check the quality of amplification and specificity. Only primers with > 90% amplification efficiency were used. The primers used are listed in Table S1.

### 2.8 Western blot

Referring to previous research (Hu et al., 2024), in brief, placenta samples were accurately weighed (<100 mg), radio immunoprecipitation assay (RIPA) lysis buffer was added at a mass: volume ratio of 1:9, and homogenized at a low temperature, centrifuged at 12,000 × *g* for 10 min at 4 °C, then the protein was denatured, and the protein concentration adjusted. The samples were run on different concentrations of sodium dodecyl sulfonate-polyacrylamide gel electrophoresis (SDS-PAGE) as needed, and then transferred to a polyvinylidene difluoride (PVDF) membrane. The blotted membranes were incubated with specific primary antibodies overnight at 4 °C and further incubated with horseradish peroxidase (HRP) bound secondary antibodies for 1 h. Binding antibodies were detected and further quantified. Antibodies were sourced from AiFang Biological. HRP-linked antibodies were purchased from Cell Signaling Technology (Danvers, MA, USA). The bound antibodies were then detected using GE ImageQuant LAS 4000 from GE Healthcare Bio-Sciences AB (Uppsala, Sweden).

### 2.9 Molecular modeling

The RES's interaction with PI3K proteins (3HIZ from PDB) was modeled using software (Molecular Operating Environment, Version, 2019).

### 2.10 The measured methods and calculation method of diets

Data was provided by China Feed Database (2020) to calculated net energy (NE) and standardized ileal digestibility (SID) amino acids. Crude protein (CP) was measured according to the GB/T 6432-2018 (China National Standard, 2018a), key instrument: VAPODEST Kjeldahl nitrogen analyzer (C. Gerhardt GmbH&Co., KG, Bonn, Germany); crude fiber according to the GB/T 6434-2022 (China National Standard, 2022), key instrument: FIBRETHERM Fiber analyzer (C. Gerhardt GmbH & Co., KG, Gerhardt, Bonn, Germany); organic matter is dry matter minus crude ash, crude ash according to the GB/T 6438-2007 (China National Standard, 2007), key instrument: muffle furnace (Jiangsu Hengli Furnace Industry Co., Ltd., Danyang, Jiangsu, China); calcium according to the GB/T 6436-2018 (China National Standard, 2018b), and total phosphorus according to the GB/T 6437-2018 (China National Standard, 2018c).

### 2.11 Statistical analysis

Results were analysed using GraphPad Prism 9 two-way ANOVA followed by Tukey's multiple comparisons test to assess the effects of HBT and RES and their interaction on reproductive performance. Statistical significance was set at *P* < 0.05.

$$Y_{ijk}=\mu +\alpha_{i}+\beta_{j}+{\left(\alpha \beta \right)}_{ij}+\varepsilon_{ijk}\text{,}$$

where *Y*_*ijk*_ is the observation value; *μ* is the overall mean; *α*_*i*_ is the level of factor A; *β*_*j*_ is the level of factor B; (*αβ*)_*ij*_ is interaction effect; *ε*_*ijk*_ is the random error term.

## 3 Results

### 3.1 Effects of RES on BT and reproductive performance in Shaziling sows

Table 2 displayed that BT in HBT sow was significantly higher than NBT sow at G75d (*P* < 0.001) and at the day of birth (*P* < 0.001), but the addition of RES in late pregnancy had limited effect on sow's BT (*P* = 0.197).

**Table 2:** Backfat thickness on G75d and effects of resveratrol (RES) on backfat thickness in Shaziling sows (mm).

Item	Groups^1^				SEM	*P*-value		
	NBT-CTL	NBT-RES	HBT-CTL	HBT-RES		HBT	RES	HBT × RES
G75d backfat thickness	22.84	22.57	27.81	27.33	0.293	<0.001	0.207	0.725
Childbirth backfat thickness	22.53	22.46	27.97	27.07	0.369	<0.001	0.197	0.263

NBT = backfat thickness of sow ranges from 20 to 24 mm; HBT = backfat thickness of sow ranges from 26 to 30 mm; CTL = fed basal diet; SEM = standard error of the mean.

*P* < 0.05 indicates significant differences, *n* = 14.

1 NBT-RES, NBT sow supplementary with 500 mg/kg RES; HBT-RES, HBT sow supplementary with 500 mg/kg RES.

As shown in Table 3, the total litter weight (*P* = 0.008), live litter weight (*P* < 0.001), average weight of total birth (*P* < 0.001), and average weight of live birth (*P* < 0.001) in HBT sows were significantly decreased compared with NBT sows. Supplementation with RES significantly increased the live litter weight (*P* = 0.009), average weight of total birth (*P* = 0.005), and average weight of live birth (*P* = 0.009). There is a significant interactive effect of HBT and RES on live litter weight (*P* = 0.018). The average process of farrowing in HBT sow was significantly prolonged compared with NBT sow (*P* < 0.001), which was significantly shortened by addition of RES (*P* = 0.009). The stillbirth rate of HBT sows was significantly increased compared with NBT sow (*P* = 0.005), the addition of RES can significantly reduce the number of stillbirths of HBT sows (*P* = 0.023). Moreover, the placental efficiency of NBT sows was significantly higher than that of HBT sows (*P* = 0.013), the addition of RES significantly increased the placental efficiency of HBT sows (*P* = 0.007).

**Table 3:** The effects of resveratrol (RES) on reproductive performance and placental efficiency in Shaziling sows.

Item	Groups^1^				SEM	*P-*value		
	NBT-CTL	NBT-RES	HBT-CTL	HBT-RES		HBT	RES	HBT × RES
Number of total births	11.21	11.07	11.07	11.14	0.550	0.948	0.948	0.846
Number of live births	10.79	10.64	9.57	10.64	0.486	0.218	0.344	0.218
Total litter weight, kg	10.45	10.62	8.74	10.03	0.415	0.008	0.084	0.182
Live litter weight, kg	10.11	10.20	7.65^b^	9.65^a^	0.387	<0.001	0.009	0.018
Average weight of total birth, kg	0.93	0.98	0.80^b^	0.91^a^	0.025	<0.001	0.005	0.222
Average weight of live birth, kg	0.94	0.97	0.81^b^	0.91^a^	0.025	<0.001	0.009	0.181
Average process of farrowing, min/piglet	14.48	13.28	18.39	16.26	0.612	<0.001	0.009	0.446
Stillbirth, %	3.41	3.30	12.71^a^	4.50^b^	1.774	0.005	0.023	0.026
Placental efficiency, w/w	5.08	5.48	4.32^b^	5.14^a^	0.215	0.013	0.007	0.345

NBT = backfat thickness of sow ranges from 20 to 24 mm; HBT = backfat thickness of sow ranges from 26 to 30 mm; CTL = fed basal diet; SEM = standard error of the mean.

*P* < 0.05 indicates significant differences, *n* = 14. The different shoulder letters on the same row indicate significant differences between NBT-CTL and NBT-RES, HBT-CTL and HBT-RES (*P* < 0.05).

1 NBT-RES, NBT sow supplementary with 500 mg/kg RES; HBT-RES, HBT sow supplementary with 500 mg/kg RES.

### 3.2 Effects of RES on serum biochemistry and inflammatory factors on the day of delivery in Shaziling sows

In Table 4, the serum level of TC (*P* < 0.001), TG (*P* = 0.010), LDL (*P* < 0.001), and MDA (*P* = 0.007) significantly increased in HBT sows. The serum level of insulin (*P* = 0.031) and T-AOC (*P* < 0.001) significantly decreased in HBT sows. The addition of RES significantly decreased the level of TC (*P* = 0.014), MDA (*P* = 0.011), increased the level of T-AOC (*P* < 0.001). And the serum level of TNF-α (*P* = 0.049), IL-1β (*P* = 0.007), and IL-6 (*P* < 0.001) significantly increased in HBT sows.

**Table 4:** The effects of resveratrol (RES) on serum biochemical indicators and inflammatory factors in Shaziling sows.

Item	Groups^1^				SEM	*P-*value		
	NBT-CTL	NBT-RES	HBT-CTL	HBT-RES		HBT	RES	HBT × RES
TC, mmol/L	0.92	0.93	1.31^a^	1.08^b^	0.044	<0.001	0.014	0.010
TG, mmol/L	0.24	0.24	0.27	0.24	0.006	0.010	0.086	0.072
LDL, mmol/L	0.22	0.22	0.27	0.24	0.010	<0.001	0.118	0.174
HDL, mmol/L	1.28	1.28	0.97^b^	1.28^a^	0.080	0.057	0.057	0.061
Glu, mmol/L	3.34	3.43	4.02^a^	3.32^b^	0.154	0.073	0.053	0.013
Insulin, pg/mL	97.84	92.90	77.90	75.94	8.288	0.031	0.679	0.858
T-AOC, μmol/mL	0.13^b^	0.17^a^	0.12^b^	0.15^a^	0.006	<0.001	<0.001	0.357
MDA, mmol/L	3.24	3.09	3.74^a^	3.26^b^	0.118	0.007	0.011	0.188
TNF-α, pg/mL	154.00	155.40	162.86	158.43	2.842	0.049	0.553	0.342
IL-1β, pg/mL	137.00	134.35	143.17	140.38	2.163	0.007	0.216	0.974
IL-6, pg/mL	168.16	168.45	189.10	181.75	3.889	<0.001	0.369	0.332

NBT = backfat thickness of sow ranges from 20 to 24 mm; HBT = backfat thickness of sow ranges from 26 to 30 mm; CTL = fed basal diet; SEM = standard error of the mean; TC = total cholesterol; TG = triglyceride; LDL = low-density lipoprotein; HDL = high-density lipoprotein; GLU = glucose; T-AOC = total antioxidant capacity; MDA = malondialdehyde; TNF-α = tumor necrosis factor-α; IL = interleukin.

*P* < 0.05 indicates significant differences, *n* = 12. The different shoulder letters on the same row indicate significant differences between NBT-CTL and NBT-RES, HBT-CTL and HBT-RES (*P* < 0.05).

1 NBT-RES, NBT sow supplementary with 500 mg/kg RES; HBT-RES, HBT sow supplementary with 500 mg/kg RES.

### 3.3 Effects of RES on placental fat deposition, oxidation, and inflammatory status in Shaziling sows

**Fig. 1:**
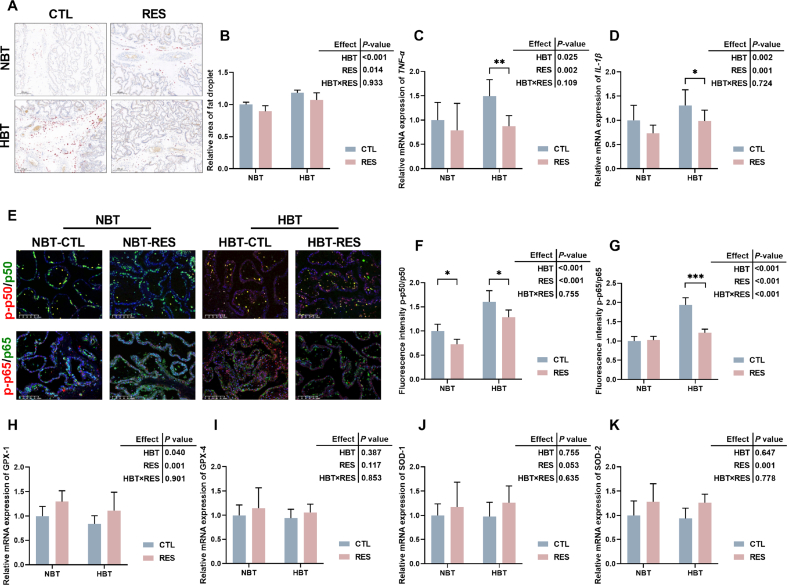
Effects of resveratrol (RES) on placental fat deposition, oxidation, and inflammatory status in Shaziling sows. (A and B) Oil red O staining of placenta tissues. The relative mRNA expression levels of *TNA-α* (C) and *IL-1β* (D). (E-G) The fluorescence intensity of p-p50/p50 and p-p65/p65 in placenta. (H) The relative mRNA expression levels of *GPX-1*, (I) *GPX-4*, (J) *SOD-1*, (K) *SOD-2*. NBT-RES, NBT sow supplementary with 500 mg/kg RES; HBT-RES, HBT sow supplementary with 500 mg/kg RES. Scale bar, 200 μm (A) and 100 μm (E). CTL = fed basal diet; NBT = backfat thickness of sow ranges from 20 to 24 mm; HBT = backfat thickness of sow ranges from 26 to 30 mm. ∗ *P* < 0.05, ∗∗ *P* < 0.01, ∗∗∗ *P* < 0.001.

The oil red O staining of placenta of Shaziling sows is shown in Fig. 1A and B, the staining degree of lipid droplets in HBT-CTL group is deeper, indicating more fat deposition in the placenta of high backfat sows, and supplementation of RES can reduce ectopic deposition of fat in the placenta.

Fig. 1C and D showed that HBT of sows affect placental inflammation status, specifically by increasing the expression of inflammation related genes *TNF-α* (*P* = 0.025) and *IL-1β* (*P* = 0.002), and adding RES reduced the expression of *TNF-α* (*P* = 0.002) and *IL-1β* (*P* = 0.001). In addition, Fig. 1E–G showed that the placental fluorescence intensity of p-p50/p50 (*P* < 0.001) and p-p65/p65 (*P* < 0.001) of HBT sows was significantly higher than NBT sows, which were reduced by addition of RES, and there is a significant interactive effect of HBT and RES on p-p65/p65 (*P* < 0.001). Fig. 1H–K showed that HBT sows had lower *GPX-1* gene expression (*P* = 0.040) in placenta, and supplementary RES increased the expression of *GPX-1* (*P* = 0.001) and *SOD-2* (*P* = 0.001).

### 3.4 Effects of RES on placental vascular and nutrient transport genes in Shaziling sows

**Fig. 2:**
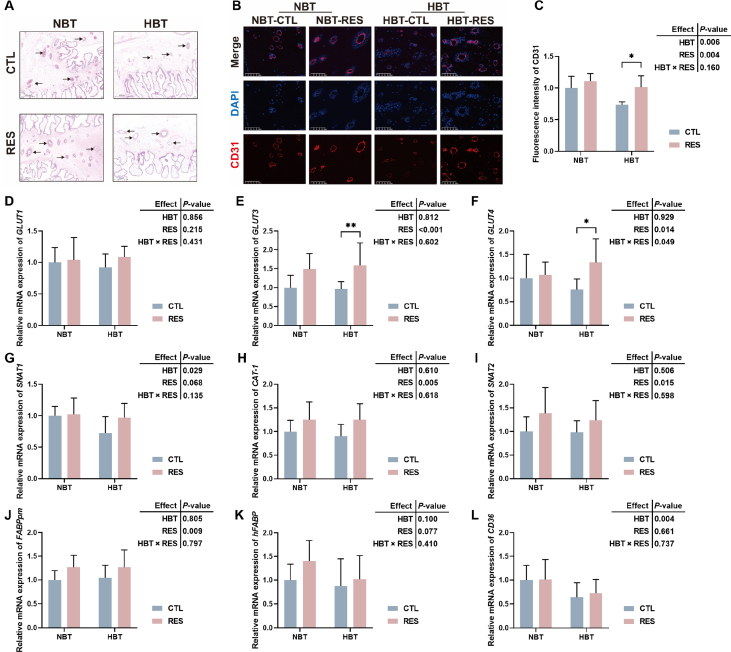
The effects of resveratrol (RES) on placental vascular and nutrient transport genes in Shaziling sows. (A) Hematoxylin and eosin staining of placenta tissues (black arrow points to the placental blood vessel). (B and C) The fluorescence intensity of cell adhesion molecule-1 (CD31) in placenta (*n* = 6). The relative mRNA expression levels of glucose transport genes (D) *GLUT1*, (E) *GLUT3*, (F) *GLUT4*, amino acid transporter genes *SNAT1* (G), *CAT-1* (H), *SNAT2* (I), fatty acid transporter genes *FABPpm* (J), *hFABP* (K), *CD36* (L) (*n* = 10). NBT-RES, NBT sow supplementary with 500 mg/kg RES; HBT-RES, HBT sow supplementary with 500 mg/kg RES. Scale bar, 200 μm (A) and 100 μm (B). CTL = fed basal diet; NBT = backfat thickness of sow ranges from 20 to 24 mm; HBT = backfat thickness of sow ranges from 26 to 30 mm; DAPI = 4',6-diamidino-2-phenylindole. ∗ *P* < 0.05, ∗∗ *P* < 0.01.

The schematic diagram of placental vascularisation is shown in Fig. 2A. The number of placental vessels was fewer in HBT-CTL group compared with NBT-CTL group, and supplementation of RES can increase the number of placental blood vessels in the field of vision. Fig. 2B and C shows the vascular markers CD31 fluorescence intensity in placenta, which in HBT sows was significantly decreased compared with NBT sows (*P* = 0.006), and significantly increased by supplementation with RES (*P* = 0.004).

Fig. 2E and F shows that adding RES in late gestation of Shaziling sows significantly increased the levels of *GLUT3* (*P* < 0.001) and *GLUT4* (*P* = 0.014) mRNA expression in placenta. As shown in Fig. 2G, the relative mRNA expression level of *SNAT1* in placenta of HBT sows was significantly decreased compared with NBT sows (*P* = 0.029), and supplementation with RES significantly increased the relative expression levels of *CAT-1* (*P* = 0.005) and *SNAT2* (*P* = 0.015) mRNA in placenta tissue (Fig. 2H and I). In Fig. 2L, the level of *CD3*6 mRNA expression in placenta of HBT sows was significantly decreased compared with NBT sows (*P* = 0.004), and supplementation with RES significantly increased the relative expression levels of *FABPpm* mRNA in placenta (*P* = 0.009) in Fig. 2J.

### 3.5 Modulation of placental angiogenesis genes and their receptors by RES in Shaziling sows

**Fig. 3:**
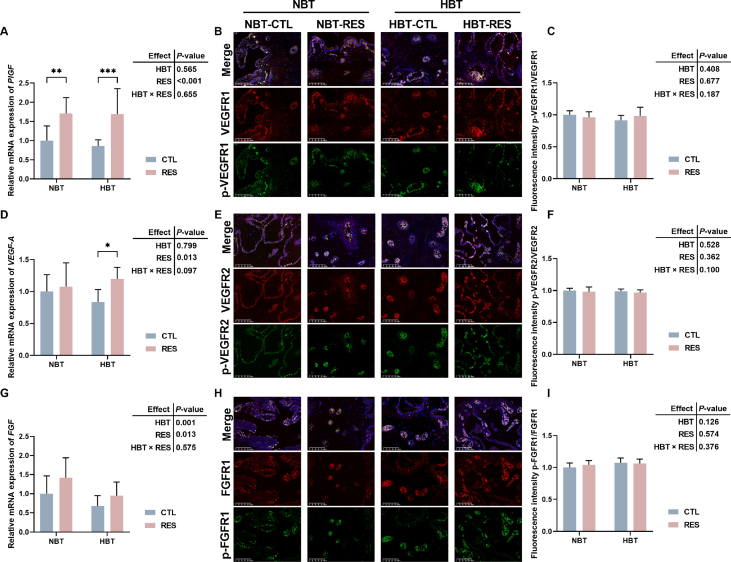
Modulation of placental angiogenesis genes and their receptors by resveratrol (RES) in Shaziling sows. (A) The relative mRNA expression levels of *FGF*. (B and C) The fluorescence intensity of p-VEGFR1/VEGFR1. (D) The relative mRNA expression levels of *PlGF*. (E and F) The fluorescence intensity of p-VEGFR2/VEGFR2. (G) The relative mRNA expression levels of *VEGF-A*. (H and I) The fluorescence intensity of p-FGFR1/FGFR1. NBT-RES, NBT sow supplementary with 500 mg/kg RES; HBT-RES, HBT sow supplementary with 500 mg/kg RES. Scale bar, 100 μm. CTL = fed basal diet; NBT = backfat thickness of sow ranges from 20 to 24 mm; HBT = backfat thickness of sow ranges from 26 to 30 mm. ∗ *P* < 0.05, ∗∗ *P* < 0.01, ∗∗∗ *P* < 0.001.

Further detection of the expression levels of angiogenetic factors related genes and their corresponding receptors’ phosphorylation. As shown in Fig. 3A–D, supplementary of RES in late gestation of Shaziling sow significantly increased the levels of *PlGF* (*P* < 0.001) and *VEGF-A* (*P* = 0.013) mRNA expression in placenta. In Fig. 3G, HBT sow had lower relative mRNA expression level of *FGF* (*P* = 0.001), which was increased by adding RES (*P* = 0.013). However, their corresponding phosphorylation of receptors did not show significant changes (*P* > 0.05; Fig. 3B, C, E, F, H, and I).

### 3.6 Resveratrol regulates placental function in Shaziling sows through the PI3K/AKT/mTOR pathway

**Fig. 4:**
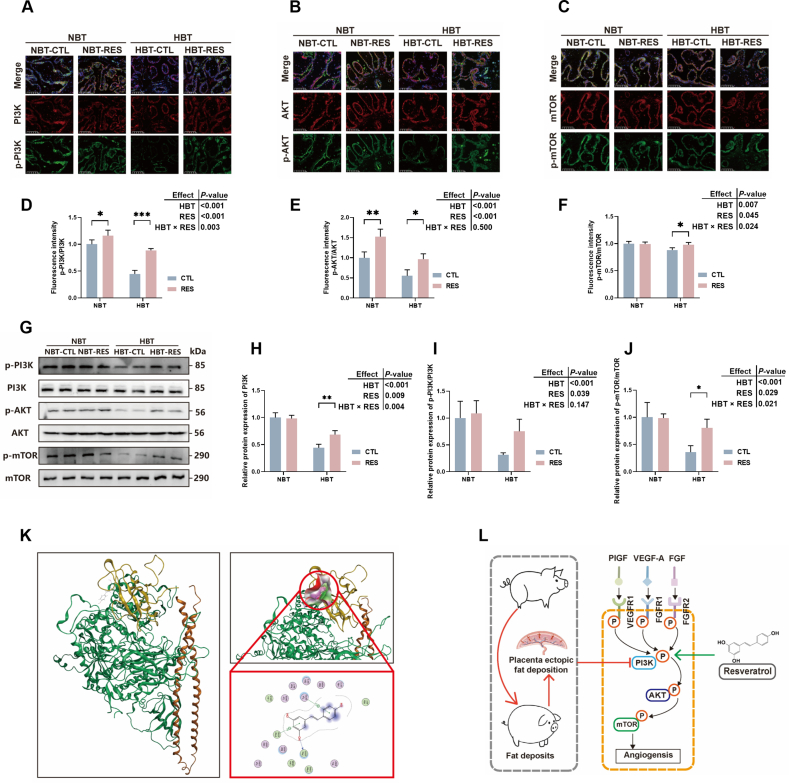
Modulation of placental angiogenesis related proteins by resveratrol (RES). (A-F) The fluorescence intensity of p-PI3K/PI3K, p-AKT/AKT, and p-mTOR/mTOR. (G-J) Protein expression levels of p-PI3K/PI3K, p-AKT/AKT, and p-mTOR/mTOR (*n* = 4). (K) The docking models of resveratrol to P85 subunit of PI3K. (L) The molecular mechanism diagram. NBT-RES, NBT sow supplementary with 500 mg/kg RES; HBT-RES, HBT sow supplementary with 500 mg/kg RES. Scale bar, 200 μm. CTL = fed basal diet; NBT = backfat thickness of sow ranges from 20 to 24 mm; HBT = backfat thickness of sow ranges from 26 to 30 mm. ∗ *P* < 0.05, ∗∗ *P* < 0.01, ∗∗∗ *P* < 0.001.

Based on the results of angiogenesis receptors had no significance between groups, to investigate whether RES directly activates the angiogenesis signaling pathway, further measured the expression of downstream proteins involved in angiogenesis. Results in Fig. 4A–F showed that HBT sows had lower fluorescence intensity of phosphorylation of PI3K (*P* < 0.001), AKT (*P* < 0.001), and mTOR (*P* = 0.007), adding RES significantly increased the fluorescence intensity of phosphorylation of PI3K (*P* < 0.001), AKT (*P* < 0.001), and mTOR (*P* = 0.045) in placenta, there is a significant interactive effect of HBT and RES on phosphorylation of PI3K (*P* = 0.003) and mTOR (*P* = 0.024). And Fig. 4G–J showed that the placental protein expression levels of p-PI3K/PI3K (*P* < 0.001), p-AKT/AKT (*P* < 0.001), and p-mTOR/mTOR (*P* < 0.001) in HBT sows was significantly lower than that in NBT sows, and RES significantly increased the expression of phosphorylation of PI3K (*P* = 0.009), AKT (*P* = 0.039), and mTOR (*P* = 0.029) in placenta, which is consistent with the fluorescence results.

PI3K can be divided into two functional subunits: P85 as the regulatory subunit, and P110 as the catalytic subunit. The phosphorylation site of PI3K is located on P110. Under normal circumstances, iSH2 structural domain (orange area) of P85 subunit binds to P110 subunit, and cannot be phosphorylated at this time, when iSH2 dissociates from P110, and the catalytic subunit of P110 can be phosphorylated. Then the computational docking analysis data in Fig. 4K shows that RES can specifically bind to SH2 domain (yellow area) of P85 (hydrogen bonding formed between RES and Pro B395), and the specific binding of this domain can promote the separation of P85 and P110, then enhance the phosphorylation ability of PI3K.

## 4 Discussion

In the swine industry, the weight condition of sows (mainly referring to BT) is an important issue considered to affect their reproductive performance. The excessive fat deposition during gestation will cause placental lipid-toxicity, chronic inflammation, and increase the level of oxidative stress in the placenta, further leading to impaired placental vascular development, an increase in weak piglets, and low litter uniformity (Kim et al., 2013; Yang et al., 2023). Maintaining an appropriate BT during pregnancy in sows is important for improving their reproductive performance. Research found that large white sows in HBT group (BT between 21 and 25 mm) have lower litter birth weight (17.17 kg) than that in medium-BT group (BT between 13 and 20 mm, 19.65 kg) (Hu and Yan, 2022). The piglet born weight and at weaning weight exhibited a quadratic pattern with the 109 d gestation BT of Yorkshire sows, and the BT between 21 to 22 mm had the highest live born weight. When the BT was ≥ 23 mm, the rate of weak piglets (piglets with weight < 800 g) increased (Zhou et al., 2018). However, compared with commercial lean type sow, there have been no reports on the relationship between BT of Shaziling sows and farrowing performance. Shaziling pig as one of “Central China Two-end-black Pigs”, have high intramuscular fat (IMF) (Song et al., 2022), high number of offspring, and long lifespan; however, lower birth weight and growth rate of piglets limit their industrial benefits. Improving the weight of piglets born alive from Shaziling sows becomes the key to promote their commercialization. This study demonstrates for the first time that Shaziling sows, as a Chinese fat-type pig, which have BT ≥ 26 mm at 75 d gestation, have poor reproductive performance, such as lower litter weight, average weight of piglets, and placenta efficiency, and RES only had positive effects on the reproductive performance of HBT sows. Excessive fat deposition at the end of gestation also causes farrowing difficulties and a greater incidence of stillborn piglets (Zaleski and Hacker, 1993). RES has been reported regulate lipid metabolism by activating phosphorylation of AMPK (Baur et al., 2006). Our previous research found that RES can activate phosphorylation of AMPK, and further upregulate peroxisome proliferator-activated receptor gamma coactivator-1 α (PGC-1α) and nuclear respiratory factor-1 (NRF-1) protein expression, alleviating uterine mitochondrial dysfunction and difficult birth caused by high-fat diets in mice (Yang et al., 2024). In this study, the HBT group had a longer average process of farrowing and higher stillbirth incidence, but this was reduced by supplementation of RES, which is consistent with previous research.

The placenta plays a key role in the communication between the mother and fetus, which is essential to fetal health and development. The expression of placental nutrient-specific transporters profoundly affects fetal weight (Brett et al., 2014). HBT in sows indicates abnormal lipid metabolism, such as disordered lipid metabolism indicators in serum (Zhou et al., 2019). The excessive fat deposition and disordered lipid metabolism leads to accumulation of ectopic fat (Wu et al., 2023a). Zhou et al. (2018) found that level of TC and HDL-C increased linearly at 109 d of gestation with the BT increasing (≤24 mm), and there was a negative correlation between the placental lipid and piglet birth weight. This study demonstrates that higher lipid accumulation in the serum and placenta in HBT sows reduced the expression of nutrient transport genes in the placenta of sows. Excessive fat deposition is converted into ceramide and diacylglycerol, further resulting chronic low-level inflammation, insulin resistance (Chaurasia and Summers, 2015), and oxidative stress (Law et al., 2018). Sows with excessive BT had higher levels of glucose, IL-6, TNF-α, and MDA in plasma, lower levels of SOD, CAT, and T-AOC in placenta compared with saws with a normal BT (Cheng et al., 2019; Hu and Yan, 2022), which is consistent with this study. Inflammation and oxidative stress have significant impacts on placental function and reproductive performance in sows. RES, a dietary polyphenol with extremely strong anti-inflammatory and antioxidant effects, effectively decreases the transcription of placental inflammatory factors, increases the gene expression of antioxidant enzymes and the transport function of placenta in this study.

Placenta tissue is rich in blood vessels, and sufficient placental angiogenesis is crucial for successful pregnancy and optimal fetal growth. Study observed that the density of vessels in placenta decreased in the low-birth-weight group (Song et al., 2018). In this study, high BT lead to a decrease in CD31 expression in placenta of Shaziling sows. Formation of new blood vessels in placenta depends on vasculogenesis and angiogenesis (Pardali et al., 2010), which is regulated directly or indirectly by VEGF, PlGF, FGF, angiopoietin-1 (Ang-1), angiopoietin-2 (Ang-2), soluble feline sarcoma (fms)-like tyrosine kinase-1 (sFlt-1), and their related signaling pathways (Huang et al., 2021; Umapathy et al., 2019). The FGF, VEGF-A, PlGF bind to FGFR, VEGFR1, VEGFR1/VEGFR2 (Simons et al., 2016), inducing receptor phosphorylation to active downstream angiogenic signals. This study shows that high BT decreased the FGF expression, and had limit effect on VEGF and PlGF, however, there were no differences in phosphorylation of related receptors, which indicates that the obstruction of placental angiogenesis in Shaziling sows is not caused by the upstream angiogenic factors and their receptors. Therefore, further focus on downstream signaling of VEGFR is required. The VEGF downstream PI3K/AKT/mTOR pathway participates in angiogenesis (Karar and Maity, 2011). PI3K mediated recruitment of AKT to phosphotyrosine-containing signalosomes has been reported as a key pathway for the development of trophoblast cells (Hu et al., 2016; Kamei et al., 2002). A previous study shows that mice with *AKT* gene disruption lead to placental insufficiency, fetal growth impairment, and neonatal mortality (Yang et al., 2003). Further, mTOR has been shown to promote endothelial cell proliferation and angiogenesis (Karar and Maity, 2011). This study indicates that high BT reduced placental angiogenesis in Shaziling sows, decreased the phosphorylation of PI3K, AKT, and mTOR in placenta. The addition of RES increased placental angiogenesis, and alleviated the inhibition of PI3K/AKT/mTOR pathway phosphorylation in HBT sows. Moreover, PI3K it was demonstrated through pull-down assay that RES can bind to P85 submit of PI3K, which promote its dissociation form P110 and phosphorylation of P110, further regulate downstream signaling pathways (Wang et al., 2020).

## 5 Conclusion

In conclusion, this study demonstrated that excessive fat deposition impaired the reproductive performance of Shaziling sows, with lower total litter weight, average weight of total births, live litter weight, average weight of live birth and placental efficiency, and had a higher average process of farrowing and stillbirth rate. However, RES can alleviate the decline in reproductive performance of Shazilng sows through regulating the angiogenesis signal pathway in placenta, and PI3K may play an important role.

## Credit Author Statement

**Xizi Yang:** Writing – review & editing, Writing – original draft, Visualization, Validation, Software, Resources, Methodology, Investigation, Formal analysis, Conceptualization. **Ruizhi Hu:** Writing – review & editing, Writing – original draft, Visualization, Validation, Software, Resources, Methodology, Investigation, Formal analysis, Conceptualization. **Wentao Zhang:** Software, Resources, Investigation, Conceptualization. **Mingkun Shi:** Software, Resources, Investigation, Conceptualization. **Zhiyong Fan:** Resources, Methodology, Conceptualization. **Xi He:** Funding acquisition. **Chenxing Fu:** Resources, Methodology, Conceptualization. **Liang Chen:** Supervision, Conceptualization. **Hongfu Zhang:** Supervision, Conceptualization. **Xupeng Yuan:** Resources, Methodology, Conceptualization. **Maisheng Wu:** Resources, Investigation. **Yulian Li:** Resources, Investigation. **Hong Tan:** Resources, Investigation. **Jianhua He:** Writing – review & editing, Project administration, Funding acquisition, Conceptualization. **Shusong Wu:** Writing – review & editing, Project administration, Funding acquisition, Conceptualization.

## Declaration of competing interest

We declare that we have no financial and personal relationships with other people or organizations that can inappropriately influence our work, and there is no professional or other personal interest of any nature or kind in any product, service and/or company that could be construed as influencing the content of this paper. Shusong Wu is an Youth Editorial Board Member for Animal Nutrition and was not involved the editorial review or the decision to publish this article.

## References

[bib1] Baur J.A., Pearson K.J., Price N.L., Jamieson H.A., Lerin C., Kalra A. (2006). Resveratrol improves health and survival of mice on a high-calorie diet. Nature.

[bib2] Brett K., Ferraro Z., Yockell-Lelievre J., Gruslin A., Adamo K. (2014). Maternal–fetal nutrient transport in pregnancy pathologies: the role of the placenta. Int J Mol Sci.

[bib3] Bustin S.A., Benes V., Garson J.A., Hellemans J., Huggett J., Kubista M. (2009). The MIQE guidelines: minimum information for publication of quantitative real-time PCR experiments. Clin Chem.

[bib4] Chaurasia B., Summers S.A. (2015). Ceramides-lipotoxic inducers of metabolic disorders. Trends Endocrinol Metabol.

[bib5] Chen C.-P., Bajoria R., Aplin J.D. (2002). Decreased vascularization and cell proliferation in placentas of intrauterine growth-restricted fetuses with abnormal umbilical artery flow velocity waveforms. Am J Obstet Gynecol.

[bib6] Chen C., Liu Y.Y., Li H.L., Zuo J.B., Yu G.J., Peng Y.L. (2022). Evaluation of muscle chemical composition, amino acids profile and antioxidative capacity of the Shaziling pig and its crossbreeds. Indian J Anim Res.

[bib7] Chen C., Zhu J., Ren H., Deng Y., Zhang X., Liu Y. (2021). Growth performance, carcass characteristics, meat quality and chemical composition of the Shaziling pig and its crossbreeds. Livest Sci.

[bib8] Cheng C., Wu X., Zhang X., Zhang X., Peng J. (2019). Obesity of sows at late pregnancy aggravates metabolic disorder of perinatal sows and affects performance and intestinal health of piglets. Animals (Basel).

[bib9] China National Standard (2020).

[bib10] China National Standard (2018).

[bib11] China National Standard (2022).

[bib12] China National Standard (2007).

[bib13] China National Standard (2018).

[bib14] China National Standard (2018).

[bib48] China Feed Database. Tables of Feed Composition and Nutritive Values in China (in Chinese). 2020. https://www.chinafeeddata.org.cn/admin/Login/slcfb [Accessed 18 December 2020].

[bib15] Gimeno-Mallench L., Mas-Bargues C., Ingles M., Olaso G., Borras C., Gambini J. (2019). Resveratrol shifts energy metabolism to increase lipid oxidation in healthy old mice. Biomed Pharmacother.

[bib16] Hsu M.H., Sheen J.M., Lin I.C., Yu H.R., Tiao M.M., Tain Y.L. (2020). Effects of maternal resveratrol on maternal high-fat diet/obesity with or without postnatal high-fat diet. Int J Mol Sci.

[bib17] Hu C., Yang Y., Li J., Wang H., Cheng C., Yang L. (2019). Maternal diet-induced obesity compromises oxidative stress status and angiogenesis in the porcine placenta by upregulating Nox2 expression. Oxid Med Cell Longev.

[bib18] Hu J., Yan P. (2022). Effects of backfat thickness on oxidative stress and inflammation of placenta in large white pigs. Vet Sci.

[bib19] Hu R., Tan J., Li Z., Wang L., Shi M., Li B. (2022). Effect of dietary resveratrol on placental function and reproductive performance of late pregnancy sows. Front Nutr.

[bib20] Hu R., Yang X., Wang L., Su D., He Z., Li J. (2024). Gut microbiota dysbiosis and oxidative damage in high-fat diet-induced impairment of spermatogenesis: role of protocatechuic acid intervention. Food Front.

[bib21] Hu X., Li J., Zhang Q., Zheng L., Wang G., Zhang X. (2016). Phosphoinositide 3-kinase (PI3K) subunit p110δ is essential for trophoblast cell differentiation and placental development in mouse. Sci Rep.

[bib22] Huang Z., Huang S., Song T., Yin Y., Tan C. (2021). Placental angiogenesis in mammals: a review of the regulatory effects of signaling pathways and functional nutrients. Adv Nutr.

[bib23] Hunan Provincial Livestock and Poultry Variety Compilation Committee (1984).

[bib24] Kamei T., Jones S.R., Chapman B.M., McGonigle K.L., Dai G., Soares M.J. (2002). The phosphatidylinositol 3-kinase/Akt signaling pathway modulates the endocrine differentiation of trophoblast cells. Mol Endocrinol.

[bib25] Karar J., Maity A. (2011). PI3K/AKT/mTOR pathway in angiogenesis. Front Mol Neurosci.

[bib26] Kim J.S., Yang X., Pangeni D., Baidoo S.K. (2015). Relationship between backfat thickness of sows during late gestation and reproductive efficiency at different parities. Acta Agric Scand A Anim Sci.

[bib27] Kim S.W., Weaver A.C., Shen Y.B., Zhao Y. (2013). Improving efficiency of sow productivity: nutrition and health. J Anim Sci Biotechnol.

[bib28] Law B.A., Liao X.H., Moore K.S., Southard A., Roddy P., Ji R.P. (2018). Lipotoxic very-long-chain ceramides cause mitochondrial dysfunction, oxidative stress, and cell death in cardiomyocytes. FASEB J.

[bib29] Mayhew T.M., Wijesekara J., Baker P.N., Ong S.S. (2004). Morphometric evidence that villous development and fetoplacental angiogenesis are compromised by intrauterine growth restriction but not by pre-eclampsia. Placenta.

[bib30] Meng Q., Guo T., Li G., Sun S., He S., Cheng B. (2018). Dietary resveratrol improves antioxidant status of sows and piglets and regulates antioxidant gene expression in placenta by Keap1-Nrf2 pathway and Sirt1. J Anim Sci Biotechnol.

[bib31] Meng Q., Li J., Wang C., Shan A. (2023). Biological function of resveratrol and its application in animal production: a review. J Anim Sci Biotechnol.

[bib32] Pardali E., Goumans M.-J., ten Dijke P. (2010). Signaling by members of the TGF-β family in vascular morphogenesis and disease. Trends Cell Biol.

[bib33] Saben J., Lindsey F., Zhong Y., Thakali K., Badger T.M., Andres A. (2014). Maternal obesity is associated with a lipotoxic placental environment. Placenta.

[bib34] Simons M., Gordon E., Claesson-Welsh L. (2016). Mechanisms and regulation of endothelial VEGF receptor signalling. Nat Rev Mol Cell Biol.

[bib35] Song B., Zheng C., Zheng J., Zhang S., Zhong Y., Guo Q. (2022). Comparisons of carcass traits, meat quality, and serum metabolome between Shaziling and Yorkshire pigs. Anim Nutr.

[bib36] Song T., Lu J., Deng Z., Xu T., Yang Y., Wei H. (2018). Maternal obesity aggravates the abnormality of porcine placenta by increasing N(6)-methyladenosine. Int J Obes.

[bib37] Umapathy A., Chamley L.W., James J.L. (2019). Reconciling the distinct roles of angiogenic/anti-angiogenic factors in the placenta and maternal circulation of normal and pathological pregnancies. Angiogenesis.

[bib38] Wang Q., Zhang P., Zhang W., Zhang X., Chen J., Ding P. (2020). PI3K activation is enhanced by FOXM1D binding to p110 and p85 subunits. Signal Transduct Targeted Ther.

[bib39] Wu D., Feng L., Hao X., Huang S., Wu Z., Ma S. (2022). Effects of dietary supplementation of gestating sows with adenosine 5ʹ-monophosphate or adenosine on placental angiogenesis and vitality of their offspring. J Anim Sci.

[bib40] Wu D., Xiong W., Ma S., Luo J., Ye H., Huang S. (2023). Konjac flour-mediated gut microbiota alleviates insulin resistance and improves placental angiogenesis of obese sows. AMB Express.

[bib41] Wu S., Tan J., Zhang H., Hou D.-X., He J. (2023). Tissue-specific mechanisms of fat metabolism that focus on insulin actions. J Adv Res.

[bib42] Yang X., Hu R., Shi M., Wang L., Yan J., Gong J. (2023). Placental malfunction, fetal survival and development caused by sow metabolic disorder: the impact of maternal oxidative stress. Antioxidants.

[bib43] Yang X.Z., Hu R.Z., Yao L.P., Zhang W.T., Shi M.K., Gong J.T. (2024). The role of uterus mitochondrial function in high-fat diet-related adverse pregnancy outcomes and protection by resveratrol. Food Funct.

[bib44] Yang Z.-Z., Tschopp O., Hemmings-Mieszczak M., Feng J., Brodbeck D., Perentes E. (2003). Protein kinase Bα/Akt1 regulates placental development and fetal growth. J Biol Chem.

[bib45] Zaleski H.M., Hacker R.R. (1993). Variables related to the progress of parturition and probability of stillbirth in swine. Can Vet J.

[bib46] Zhou Y., Xu T., Cai A., Wu Y., Wei H., Jiang S. (2018). Excessive backfat of sows at 109 d of gestation induces lipotoxic placental environment and is associated with declining reproductive performance. J Anim Sci.

[bib47] Zhou Y., Xu T., Wu Y., Wei H., Peng J. (2019). Oxidative stress and inflammation in sows with excess backfat: up-regulated cytokine expression and elevated oxidative stress biomarkers in placenta. Animals (Basel).

