# Supplementary material for: Effect of fat deposition on placental function in Shaziling sows and modulation by resveratrol
Source: Anim Nutr. 2025 Dec 6;24:368–77. doi: 10.1016/j.aninu.2025.08.009 (PMC12914803; doi:10.1016/j.aninu.2025.08.009)
Supplement: Multimedia component 1 [file mmc1.docx]

Table S1 Primers used for real-time PCR.

| Genes | Full names | GenBank number | Sequences | Size, bp |
| --- | --- | --- | --- | --- |
| β-Actin |  | NM_001170517.2 | F: 5’ TCTGGCACCACACCTTCTACAAC 3’  R: 5’ GTCATCTTCTCACGGTTGGCTTTG 3’ | 106 |
| *GADPH* | Glyceraldehyde 3-phosphate dehydrogenase | NM_001206359.1 | F: 5’ GGCTGTGGGCAAGGTCATCC 3’  R: 5’ GTTTCTCCAGGCGGCAGGTC 3’ | 110 |
| *GLUT1* | Glucose transporter protein 1 | XM_021096908.1 | F: 5’ CCTGGTCCTGTTCTTCATCTTCAC 3’  R: 5’ CTCGGGTGTCTTGTCGCTTTG 3’ | 121 |
| *GLUT3* | Glucose transporter protein 3 | XM_021092391.1 | F: 5’ GCTACAACACTGGAGTCATCAATGC 3’  R: 5’ GCCACAGACAAGGACCACAGG 3’ | 130 |
| *GLUT4* | Glucose transporter protein 4 | NM_001128433.1 | F: 5’ TCAACACAGTCTTCACCTTGGTCTC 3’  R: 5’ GCGGAAGCAGAGCCACAGTC 3’ | 126 |
| *SNAT1* | Sodium-coupled neutral amino acid transporter 1 | XM_003355629.4 | F: 5’ GAAAGCAGAAGAAGTCTCACGA 3’  R: 5’ CAAAGGCGAGTCCCAGAAT 3’ | 142 |
| *CAT-1* | Catalase-1 | XM_021065162.1 | F: 5’ ACATCTCAACCAGCCTCATAGCG 3’  R: 5’ GCCGTGACCACCAAGCAGAG 3’ | 142 |
| *SNAT2* | Sodium-coupled neutral amino acid transporter 2 | NM_001317081.1 | F: 5’ CGCAGCCGTAGAAGAATGATGAATG 3’  R: 5’ GGTGTGAAGCAATTCCGTCTCAAC 3’ | 129 |
| *FABPpm* | Plasma membrane fatty acid binding protein | NM_213928.1 | F: 5’ GCTTTGACTTCACAGGTGCTCTTG 3’  R: 5’ GCCATCTCCTTCCACTGCTCAG 3’ | 127 |
| *hFABP* | Heart type fatty acid binding protein | NM_001099931.1 | F: 5’ AGCACCTTCAAGAGCACAGAGATC 3’  R: 5’ TGCCTCCATCCAGTGTCACAATG 3’ | 106 |
| *CD36* | Cluster of differentiation 36 | NM_001044622.1 | F: 5’ ACAGTCTCTTTCCTACAGCCCAATG 3’  R: 5’ TGCCACAGCCAGATTGAGAACAG 3’ | 99 |
| *FGF* | Fibroblast growth factor | XM_013987618.2 | F: 5’ CCAGGAAATTACAAGAAGCCCAAGC 3’  R: 5’ GGTCCCATCCACTGTGCCATC 3’ | 90 |
| *PlGF* | Placental growth factor | XM_003125167.6 | F: 5’ TCAAGAGACTGCTGTATGCCCATC 3’  R: 5’ ACAACCATGTCAAGTGCGTTTCC 3’ | 117 |
| *VEGF-A* | Vascular endothelial growth factor-A | NM_214084.1 | F: 5’ GGCAGAAGGAGACCAGAAACCC 3’  R: 5’ ACAGGACGGCTTGAAGATGTACTC 3’ | 142 |
| *TNF-α* | Tumor necrosis factor-α | NM_214022.1 | F: 5’ CCACCACGCTCTTCTGCCTAC 3’  R: 5’ TTGAGACGATGATCTGAGTCCTTGG 3’ | 116 |
| *IL-1β* | Interleukin-1β | NM_001302388.2 | F: 5’ GTGTCTGTGATTGTGGCAAAGGAG 3’  R: 5’ AGGACGATGGGCTCTTCTTCAAAG 3’ | 113 |
| *GPX-1* | Glutathione peroxidase-1 | NM_214201.1 | F: 5’ CTCACCCGCTCTTCGCCTTC 3’  R: 5’ TCATTGCGACACACTGGAGACC 3’ | 112 |
| *GPX-4* | Glutathione peroxidase-4 | NM_214407.1 | F: 5’ CCTCATTGATAAGAACGGCTGTGTG 3’  R: 5’ GCACGGCAGGTCCTTCTCTATG 3’ | 82 |
| *SOD-1* | Superoxide dismutase-1 | NM_001190422.1 | F: 5’ ATCAAGAGAGGCACGTTGGAGAC 3’  R: 5’ CCGAGAGGGCGATCACAGAATC 3’ | 96 |
| *SOD-2* | Superoxide dismutase-2 | NM_214127.2 | F: 5’ TCTGGACAAATCTGAGCCCTAACG 3’  R: 5’ ACGGATACAGCGGTCAACTTCTC 3’ | 118 |

F = forward; R = reverse.
